# On a New Remedy for Dysentery

**Published:** 1865-10

**Authors:** William Kerr

**Affiliations:** Surgeon, &c., &c.


### ON A NEW REMEDY FOR DYSENTERY.

By WILLIAM KERR, Surgeon, &c., &c.

Twelve years ago, an accidental circumstance led me to attempt an improvement in the treatment of dysentery. Commencing with camphor and henbane, added to opium, I experimented on every officinal narcotic, coming to the conclusion, that of these the most efficient combination was one of opium, henbane, hemlock, stramonium, and digitalis. I had cause to be better satisfied with this than with any previous combination; but from time to time failures or tardy success induced the conclusion that something was still wanting—that something, if to be found at all, was therefore to be discovered in plants not yet admitted into the Pharmacopœias. After a long search, cicuta maculata, sium lineare, and conio-selinum canadense, indigenous to the swamps and woods of Canada, supplied the deficiency better than any others I happened to try. Sium lineare supplanted hemlock (conium maculatum), on account of the combination containing the latter occasionally producing pain in the bowels and failing, while that with sium lineare gave relief; and dulcamara supplanted henbane, as experience showed it to be better adapted to act beneficially along with the other members of the combination. Its constituents, when the investigation was concluded, were as follows:—four officinal, viz., opium, stramonium, dulcamara, digitalis; three non-officinal, sium lineare, cicuta maculata, conio-selinum canadense. All are more or less narcotic; and digitalis, dulcamara, and sium lineare are also diuretic. So many are necessary evidently, from each possessing some peculiarity in the way in which it affects the system: the combined effect of these peculiarities being required to combat the disease.

Without opium the combination is slightly aperient, improves appetite, promotes sleep, and, according to experience gained in dysentery and other diseases, heals ulceration of the mucous membrane. In dysentery, opium is necessary apparently to check the frequent motions of the bowels, the strictly curative power depending chiefly, if not altogether, on the other ingredients. In infants generally, and also in a few adults, digitalis does not act favorably. In such instances I have substituted squills with great benefit. Adults generally require the combination with digitalis; of a very few infants the same may be said; and to many adults the combination with digitalis, or that of squills, may be given indifferently. Excepting opium, the part employed is leaf. Digitalis and squills are combined in the proportion of half a part of each—all the others in that of one part. For infants, opium is reduced to a half part. The usual dose to adults is six and a half grains, digitalis or squills being each half a grain, and all the others one grain each.

Between five and six years were spent in determining the components. Beginning with three, I never afterwards, either in adding or subtracting, changed more than one plant, till I had as fully as lay in my power ascertained the result of each change. In this manner I have experimented on thirty-two plants or their products. I have pulled down the combination and built it up again, and thus done my best to ascertain the necessity for each component. For upwards of seven years the combination has been used with very great success; but as my own experience may be suspected of being biassed, I shall confine myself to the reports of others.

Dr. Brown, of Berlin, C. W., had a very severe attack, of which he published an account in the *Montreal Medical Chronicle* for December, 1858.

Of this paper the following is a copy, slightly abridged:— "In August last, I was seized with epidemic dysentery. The usual remedies were promptly administered—opium, the quantity of which speedily rose to twenty-one and even twenty-four grains daily, together with mercury, acetate of lead, and ipecacuanha, but no amendment took place. I vomited incessantly, and, though tormented with thirst, could retain no fluid. In my case the effect of large doses of opium was prostrating and overpowering. I did not sleep, but could scarcely be said to be awake, except to the consciousness of severe pain, agonizing tenesmus, and frequent vomiting. I had been ten days ill, nature was sinking, collapse was to be feared, when Dr. Kerr visited me. He immediately gave three and a half grains, or half a grain of each of the seven ingredients." (The recipe is here given by Dr. B.)

"I was very restless from a sensation of sinking and severe pain. In half an hour, after dozing a few minutes, I became aware of a great change. I could lie quiet; the distressing tenesmus was less, pain in the body and limbs less severe, the sensation of sinking relieved, a glow of warmth was supplanting the cold of threatened collapse, and an inclination to sleep, not before experienced during my illness, was stealing over me. The first thought was amazement at the change, then a faint recollection of a new medicine crossed my mind, and I resigned myself to its influence. I was immediately asleep, and for an hour and a-half had a comfortable and refreshing sleep, unaccompanied, comparatively speaking, with sensorial disturbance. When I awoke all the symptoms were relieved. Seven grains were given every six hours; but the quantity of digitalis being too great, this drug was reduced from a full to a-half proportion, making each dose six and a-half grains, which were given every four hours.* I spent twenty-four hours almost wholly in sleep; calls to rise were still frequent, but the tenesmus was less severe, and, though I retched a few times, vomiting ceased. In a few days appetite began to return.

"During twelve years' practice, I never, in the treatment of dysentery, met with a narcotic to be compared with Dr. Kerr's combination, in relieving general irritability, pain, and, above all, nausea and vomiting. It produces a wonderful degree of comfort, unattended by sensorial disturbance. From thirty minutes after the first dose was taken, my suffering was comparatively nothing. Little hope was entertained of my recovery previous to the first dose, but became sanguine before I had taken the third."† Dr. Bingham, Dr. Brown's medical attend-

* Prior to this all the ingredients were equal.

† The possibility of any future report from Dr. Brown was cut off by his accidental death a few months afterwards.

ant, in a supplement, vouches for the accuracy of the narrative, and relates six confirmatory cases from his own experience.

Dr. Bingham, supplied with medicine by me, treated successfully the sporadic cases which occurred in the following years:—In August, 1862, he, with Dr. Bell, by this time his partner, applied to me, making the following statement:—Dysentery had broken out epidemically in their locality, but not having any of my medicine, they had treated it with the usual remedies; a woman had died the preceding evening, her husband was dangerously ill, and other two were apparently dying. Furnished with a supply, they hastened to their patients. The husband just mentioned, though previously ill for five days, was relieved in less than an hour, and had a rapid recovery. One of those believed to be dying recovered readily, though upwards of seventy years of age; the other died, time to administer only a single dose being afforded. During the remainder of the epidemic there was not a death, though, judging from the severity of the attacks, six or seven would have proved fatal under the ordinary treatment. In the autumn of 1863, dysentery was again epidemic at Ayr, C. W., where Drs. Bell and Bingham resided. Without delay they applied to me for medicine, and treated successfully every case; while the only other medical gentleman in the same village adhered to the ordinary treatment, and out of a smaller number of patients lost five by death.

Dr. Mackintosh, of Hamilton, C. W., has employed the combination in dysentery since 1861, and in all cases with success. From his notes I give the following, on account of the epidemic and generally severe character of the attacks:—

"1864, 15th July.—A child, four years of age, seized two days ago, bloody stools every half hour, accompanied with vomiting and severe pain. Applied hot fomentations, and gave three grains of the squill combination with opium every four hours. These were speedily followed by relief; the child had a pretty good night, and on the 19th is reported quite well.

"17th July.—A girl in the same house, nine years of age, was seized during the night with severe dysentery. Applied hot fomentations, gave five grains of the digitalis combination with opium every three hours. Immediate relief followed, and next day she was convalescent. Six doses in all were given.

"22d July.—A boy, seven years of age, seized yesterday, and now severely affected. Gave three grains every three hours, and by evening he was much relieved. Nine doses completed the cure.

"6th August.—A man, aged thirty, attacked during the night with rigors and vomiting, followed by dysentery. In the morning seven grains were given every three hours. In two days he was quite well.

"12th August.—A man, aged sixty-four; bloody motions every half hour, with nausea. Same doses given as to last patient; next day almost well.

"15th August.—A woman, aged fifty-six, during the night was attacked with severe dysentery. Same doses given. By evening was much better; and by the third day complained merely of weakness.

"17th August.—A woman, suddenly seized with very severe dysentery, visited shortly after; she was then cold and faint, and stools passed without control. Gave seven grains of the digitalis combination, with one-fourth of a grain of morphia (instead of opium), every two hours. After the third dose relief was so great that morphia was altogether omitted, but seven grains of the other combination were continued thrice a day.

"19th.—Almost well."

Dr. Philip, of Galt, late assistant-surgeon H.M. 18th Regiment, has furnished me with the following statement:—"Your remedy was administered by me in six severe, besides a number of slighter cases of dysentery, during the autumn of 1862. Relief was uniformly obtained after one to two doses, and recovery completed within a few days. One of the cases was characterized by profuse sanguineous discharge, and, occurring in a delicate female, would probably have proved fatal but for the timely administration of this medicine. In contrasting the success of treatment in these instances with the fruitless and unfortunate attempts made by myself and others at relief in the severe dysentery of the Crimea, it is impossible not to be struck with the readiness and efficacy of this remedy. Every known system of treatment, I believe, was tried, and the medical history of the compaign shows with how little benefit. Many of the cases which in the Crimea ended fatally were not apparently of a more severe character than some of those which yielded rapidly to your medicine."

Dr. Merritt, who at the time the following occurred was chief of the Medical Department of the Confederate Army of the Mississippi, thus writes to me:—"In August, 1863, when in charge of Camp Jackson, I came into possession of a quantity of your medicine for dysentery. The rapidity of relief and of cure was exceedingly striking. The men were on their feet in a few days, and in worst cases I did not give more than eight doses of six grains each. My supply lasted ten days, and was administered to about sixty patients, only one of whom died. For some time before I obtained the medicine the deaths ranged from one to three daily, and as soon as it was all expended the mortality resumed the same rate."

A child of the Rev. Mr. Robb, Calabar, Western Africa, in the latter part of July, 1863, was seized with dysentery. At this time the favorite treatment at the mission was large doses of ipecacuan; but this illness resisted every prescription of Dr. Hewan, the medical attendant. By the middle of August the child became so reduced, and death so impressed on the visage, that recovery was regarded by all to be hopeless. At this juncture the parents recollected a packet of the combination which I had given to them. After the second dose the child awoke from a refreshing sleep, easy and tranquil, and the medicine being continued, recovery went on rapidly, without a single untoward symptom. A second attack of dysentery, a few months afterwards, was stopped in a single day. A native African was cured of what threatened to be a severe illness by ten doses.

Fifteen medical men besides myself have used this combination in dysentery; it has been given in the warm region of California, amid the privations and discomforts of a camp in the hot summer of the Southern States, at sea on the Atlantic, in the tropical and pestilential climate of Calabar, and there is a remarkable uniformity in the testimony of all. Relief generally in an hour, restoration to health in a few days, and the great majority cured within a week. A few cases were a little tedious, and a still smaller number lingered three or four weeks; none lapsed into chronic dysentery; and out of about *four to five hundred patients*, though several of the attacks were very severe, as severe as some of the reporters had ever witnessed, *only four died.* One of these was a delicate child; the second, an infant on whom the medical attendant had previously exhausted all the ordinary medicines; and the third and fourth have not been specially reported to me. I have been told of some instances, and a few have occurred in my own practice, of that generally fatal variety of dysentery characterized by profuse bloody discharges, usually attended with severe pain, all of whom were cured without difficulty. The combination fails in chronic diarrhœa, possibly because this disease is usually unaccompanied by lesion of the mucous membrane. I have not seen or had reported to me any disagreeable effect from this remedy, though, judging from the character of its constituents, such is possible, were the doses unreasonably large. The *medicinal* power is certainly greatly increased by the combination, but not the *poisonous.* Relief speedy and great of pain, far sounder and more refreshing sleep than that from opium, and cessation of discharges, are the usual effects. The nearly uniform success has not given either my correspondents or myself opportunities of trying the treatment by large doses of ipecacuan.

In the course of my experience, several persons afflicted with chronic dysentery have been restored to health—some by the combination containing opium, others by that without. Dr. Ogden, lecturer on materia medica, Toronto, tells me of a case of acute dysentery where, from idiosyncrasy, opium disagreed, but which was speedily cured by the combination, leaving out this drug.

Cases of cholera infantum have been reported to me by medical friends as treated successfully by the combination containing opium. Of summer cholera I select the following on account of its severity. A young man was seized during the night, and visited by Dr. Bingham in the morning. At this time he was violently cramped, skin cold and clammy, voice husky, and pulse feeble. Eight grains of the combination containing opium were given; from this time he vomited no more, a glow of warmth (as in Dr. Brown's case of dysentery) supplanted the cold of threatened collapse, and cramps rapidly abated in severity, though all day he had muscular twitchings. Four more doses completed the cure. Summer cholera has been for some years a rare disease, but all treated by my medical correspondents or myself have readily recovered.

In conclusion, I beg to suggest the medicine now recommended as worthy of trial in Asiatic cholera. If a remedy for this disease is ever to be found, it clearly must be one which shall take almost immediate effect.—*Edinburgh Med. Journal.*

*Doon Grange, near Galt, Canada West, 5th April,* 1865.

